# Association between water, sanitation, general hygiene and oral hygiene practices of street-involved young people in Southwest Nigeria

**DOI:** 10.1186/s12903-020-1022-z

**Published:** 2020-01-31

**Authors:** Morenike Oluwatoyin Folayan, Mary O. Obiyan, Atinuke O. Olaleye

**Affiliations:** 1 Department of Child Dental Health, Obafemi Awolowo University, Ile-Ife, Nigeria; 2 Department of Demography and Social Statistics, Obafemi Awolowo University, Ile-Ife, Nigeria; 3 Department of Obstetrics and Gynecology, Babcock University, Ilishan, Nigeria

**Keywords:** Oral hygiene, Sanitation, Homeless youth, Hygiene, Nigeria

## Abstract

**Background:**

Oral hygiene practices can be linked to personal hygiene practices, including access to water and other sanitation facilities. The objective of the study was to determine if there is an association between oral hygiene practices and water and sanitation hygiene (WASH) practices among street-involved young people (SIYP).

**Methods:**

A cross-sectional study recruited SIYP age 10–24 years in two States in Nigeria recruited through respondent-driven sampling in December 2018. Interviewer-administered questionnaires were used to collect data on water access, sanitation, personal and oral hygiene. The instruments used for collecting the data were standardized tools for measuring the phenomena studied. The association between knowledge and practice of oral hygiene; oral hygiene and water, sanitation and hygiene (WASH); and indicators of good oral hygiene were determined using binary logistic regression guided by two models.

**Results:**

A total of 845 study participants were recruited. The proportion of SIYP with good knowledge of oral hygiene was low (31.2%), and fewer had good oral hygiene practice (8.9%). There were significant associations between knowledge and practice of tooth cleaning, use of fluoride-containing toothpaste, dental flossing, consumption of sugar between meals, and frequency of dental check-ups (*p* < 0.001 respectively). Respondents with good water collection and storage practices (AOR: 2.01; 95% CI: 1.24–3.24; *P* = 0.005) and those residing in Lagos (AOR: 2.85; 95% CI: 1.61–5.06; *P* = 0.001) had a higher likelihood of having good oral hygiene.

**Conclusion:**

Good oral hygiene practices of SIYP in Nigeria is associated with access to water collection and storage. WASH programs can have an impact on health through improved oral hygiene practices.

## Background

Access to water is strongly associated with hygiene practices, and hygiene practices are strongly linked to health. For these reasons, one of the 2030 Agenda for Sustainable Development goals is access to safe drinking water. Safe water can be a driver of progress for many of the Sustainable Development goals including health, nutrition, education and gender equality [1]. This was a reason for global actions to improve water access, sanitation and hygiene (WASH). The aim of WASH is to empower communities to enhance their access to and use of safe water and sanitation service, and to improve sustainable hygiene practices [2].

Such access should improve oral hygiene practices also as oral hygiene is a part of personal hygiene. Personal hygiene in the context of the WASH initiative does not directly address oral hygiene [3]. Yet, poor oral hygiene contributes to poor general health [4]. Poor oral hygiene.

habits and practices are established early in life [5] and are associated with increased risk for diseases in adulthood [6]. Good oral hygiene practices on the other hand, are associated with knowledge of what constitutes good practices [7].

Though the ability to self-regulate improves during adolescence, the motivation to employ self-regulatory abilities for health-related practices declines during this period with implications for overall oral health and well-being [8, 9]. This decline may be worse for adolescents and youths who live without parental influence and control, such as street-involved young persons (SIYP).

SIYP are adolescents and young persons who come to the street and engage in the street economy as a source of labor [10]. They also engage in high-risk behaviors [11] and have emergency health needs [12]. In Nigeria, the risk for sexually transmitted infection, HIV infection and sexual exploitation are increased in SIYP [13–16]. SIYP often reside in slums and poor residential areas [12], which increases their risk for poor sanitation and personal hygiene and oral health practices [17–19].

Though oral hygiene is part of personal hygiene practices, oral hygiene is at the greatest risk of neglect by children, adolescents and adults when faced with multiple life issues [20]. Those in the labor industry (SIYP who also are working agents) often de-prioritized oral health practices in comparison to other personal hygiene practices [21].

WASH are country-level programs that aim to reduce the risk for diseases by creating environments that support good personal hygiene and sanitation. Little is known about the link between WASH practices and oral health. Yet, with the high prevalence of dental caries in developing countries [22] - the same regions where WASH programs are implemented and expected to have a major impact - incorporating oral hygiene practices into WASH programs may be a cost-effective approach to addressing important health needs at the population level. WASH programs have focused on under-5-year age children to reduce the risk of childhood infectious diseases that result from inadequate access to water and sanitation [23]. Though there is a WASH agenda for Nigeria [24], our literature search yielded no publications on WASH and its association with SIYP practices and oral health practices.

Poor oral health is perpetuated by social inequality just like poor access to water, sanitation and personal hygiene [25]. Oral hygiene practices can be affected by demographic factors (age, sex, education, and employment), distal factors (access to water and sanitation services), and proximal factors (oral health knowledge). This study will determine the association between the knowledge and practice of oral hygiene by SIYP in Nigeria; and the association between oral hygiene practices and other personal hygiene practices (access to water and other sanitation facilities). We hypothesize that poor oral hygiene practices are associated with poor access to water, sanitation and personal hygiene for SIYP. We also hypothesize that personal hygiene practices are strongly associated with access to water and access to other sanitation facilities [7].

## Methods

### Study design and study population

This was a cross-sectional study that explored the knowledge and hygiene practices of SIYP in southwest Nigeria. A community engagement program (CEP) on WASH was implemented in December 2018, as a SIYP community entry strategy with the aim of fostering rapport between the field workers and study participants. This approach was adopted to facilitate and retain participation of SIYP for a one-year project that commenced after the CEP program. The WASH CEP consisted of health talks on personal hygiene (hand-washing, sanitation, oral hygiene, and menstrual hygiene) and street hygiene. Data were collected from recruited SIYP before the WASH CEP began. Study participants were adolescents and young people, aged 10 to 24 years, who met the criteria for being SIYP (those who spend most of their time on the street and return home at night, or who continually live and sleep on the street).

### Sample size

Sample size determination for the project was guided by Turner [26], who recommended estimates to derive sample size for surveys on orphaned and vulnerable children (OVC) in homeless situations. Considering the unavailability of data to generate prevalence rate of OVC in the proposed study environment, the suggested minimum sample size of 800 to 1000 was adopted for this study. A total of 845 (452 males and 393 females) SIYP were interviewed during the WASH CEP.

### Study instruments

The WASH questionnaire had sections on socio-demographic characteristics, personal hygiene (collection and storage, handwashing, sanitation and waste disposal), knowledge about hygienic practices, and oral hygiene. Each section was adopted from validated sources. The tool used to collect information on personal hygiene was adapted from the United Nations High Commissioner for Refugees WASH knowledge, attitude and practice survey questionnaire [27]. The tool used to collect data on oral hygiene has been used in past studies in Nigeria [28]. A national WASH consultant who is a community health expert in sanitarian and public health undertook the judgment quantification of the instruments used for the study. The judgement quantification is a part of the instrument content validation process [29]. A pilot was then conducted to test the questions for ease of understanding and language clarity. The survey took place between the first and third weeks of December 2018.

*Socio-demographic data* collected were age, sex, marital status, school attendance and state of residence. Age was designated as age at last birthday and was stratified into three groups: 10–14 years, 15–19 years, and 20–24 years. Marital status was designated either married or not married; those living with their spouse were considered married. The response on the history of school attendance was either ‘yes’ or ‘no’. A ‘yes’ response indicated that the person had had some level of formal education.

*Information on oral hygiene practices* was collected with a 5-item questionnaire that sought information on the frequency of tooth cleaning, use of fluoride-containing toothpaste, use of dental floss, consumption of sugar between meals, and frequency of dental check-ups. The responses to the questions ranged from four to seven alternatives. The accepGuidelines for Sampling Orphans and Other Vulnerable Children responses were brushing more than once a day, use of fluoridated toothpaste always or almost always, flossing at least once a day, eating sugary snacks between main meals less frequently than once a day, and attending a dental check-up within the last year. Only three of the five items were used to measure good oral hygiene practice. The expected practices were: brushing teeth more than once a day, used fluoridated toothpaste always or almost always, and ate sugary snacks between main meals less often than once a day [28]. The responses were given a score of 1 and otherwise 0. A possible score range of oral hygiene practices was 0–3. Respondents were considered to have good oral hygiene practice [24] if they had 2 to 3 of these oral health practices: Those with 0 to 1 practice were considered to have poor oral hygiene practice.

### Information on oral hygiene knowledge

The questions on oral hygiene practices were adapted to inquire about respondents’ oral hygiene knowledge. Respondents were asked about their knowledge of the frequency of tooth cleaning, use of fluoride-containing toothpaste, use of dental floss, consumption of sugar between meals, and frequency of dental check-ups. Correct responses to the questions were assigned ‘1’ if positive and ‘0’ if otherwise. After that, an index was computed for the variables used to measure oral hygiene knowledge with a possible score ranging from 0 to 5. The mean score cut-off point was 2. Respondents who answered yes to 3 and more of the 5 questions reflected good oral hygiene knowledge. Responses fewer than these three correct responses were categorized as poor oral hygiene knowledge [30]. The questions on oral hygiene knowledge were asked after the questions on oral hygiene practices.

### Information on personal hygiene

There were four questions on personal hygiene: water collection and storage, handwashing, sanitation and waste disposal. The section on water collection and storage had 5-item, closed-ended questions that explored responses about source of drinking water (improved and non-improved); and water storage (if had containers for collecting and storing water; if the container for collecting and storing water is protected; frequency of cleaning drinking and storage water containers and how the containers were cleaned). Those who had good ‘water collection’ and ‘storage practice’ were assigned ‘1’ while others were assigned ‘0’.

For handwashing practices, a 4-item close-ended questionnaire was used to elicit information on washing hand with soap, washing hands before eating, washing hands after toilet, and washing hands soon after getting home. Responses to the questions on handwashing practices were coded as no - ‘0’and yes – ‘1’. The possible score range was 0 to 4. Only respondents who answered positive to the four questions were assumed to have good handwashing practices.

A 4-item questionnaire was used to elicit information on sanitation. It obtained information on the usual place for defecation (improved facility and non-improved facility); access to handwashing facility at a place of defecation and access to water and soap to wash hands after defecation. The responses to the questions on sanitation were coded as ‘0’ – no and ‘1’ – yes and were thereafter merged. The score for good sanitation practice ranged from 0 to 4. Any score less than 4 was categorized as poor sanitation.

A 3-item questionnaire was on the knowledge and practices of management of waste. It inquired about methods for disposal of waste (recycle, reuse, dispose in waste bin, dump in the bush), if respondent considered it safe to use of bare hands to collect waste (yes, no, don’t know), and if aware that improper management of wastes pollutes water and causes typhoid fever (yes or no or don’t know). Good knowledge and practice of waste management was scored ‘1’ and poor knowledge and practice of waste management was scored ‘0’. All ‘don’t know’ responses were scored ‘0’. The possible score for good waste disposal ranged from 0 to 3. Any score less than 3 was categorized as poor waste management.

### Study procedures

The study participants were recruited through respondent-driven sampling [31]. The first seeds for the respondent-driven sampling were identified in areas where SIYP cluster. Clusters were identified through mapping conducted by the research team along with officials of the State Ministry of Health. Two high-volume clusters each in Lagos and Osun States were identified: the cluster sites in Lagos State were Bariga and Ajah; the sites in Osun State were Oke-Baale and Olaiya/Sabo.Two cards with identification numbers were given to the first ten seeds in these cluster areas after they had been interviewed to recruit friends or peers, which some SIYP accomplished.

The recruitment effort of seeds was complemented by direct recruitment by the research team, who also visited the cluster areas. When SIYP approached for study participation were not willing to participate, field workers went on to recruit the next possible SIYP. Recruitment continued until at each cluster until target sample size were reached.

All recruited study participants gave consent before administration of the questionnaire by research team members. The questionnaire was administered in a place the respondent felt most comfortable to respond to questions. At the end of the interview, participants were given lottery tickets that enabled them to enter a raffle draw during a sideshow at the end of the survey. The values of the draw prize were $0.40 to $2.25.

### Variables

The outcome variable for this study was ‘oral hygiene practices,’ measured as either good or poor. The explanatory variables were good or poor water collection and storage, handwashing, sanitation and waste disposal practices. Confounding variables were the background information of respondents: sex, age, state of residence, school attendance, and marital status.

### Data analysis

Data were collected electronically with open data kit, and analyzed using STATA 15.1. Univariate analysis was conducted to describe the proportion of respondents for the outcome, explanatory and confounding variables. Bivariate analysis was conducted with Pearson chi-square (or Fisher’s exact t-test where appropriate) to determine the associations between the outcome variables and the explanatory and confounding variables. Inferential analysis was conducted to identify risk indicators for good oral hygiene. Binary logistic regression, guided by two models, was conducted. The first model tested the association between the outcome and explanatory variables while the second model tested the associations between outcome and explanatory variables adjusting for confounders. Statistical significance was considered at *p*-value less or equal to 0.05.

## Results

### Oral hygiene knowledge

More than half of the respondents had good knowledge about the frequency of tooth cleaning (52.4%) and were aware of the need to use fluoride-containing toothpaste (62.4%). However, a low proportion of respondents had good knowledge on the frequency of use of dental floss (22.2%), frequency for consumption of sugar between meals (15.5%), and frequency of dental check-ups (31.6%). Two hundred and sixty-four (31.2%) SIYP had good oral hygiene knowledge. Table 1 lists the proportion of respondents for each item of the oral hygiene knowledge assessed.

**Table 1:** Oral hygiene knowledge of SIYP in Lagos and Osun State, Nigeria (*N* = 845)

Variables	Sex		Age			Residence		Total *N* = 845
	Male (*n* = 452)	Female (*n* = 393)	10–14 years (n = 229)	15–19 years (*n* = 372)	20–24 years (*n* = 244)	Osun (*n* = 417)	Lagos (*n* = 428)	
Sugar intake in-between meals								
Rarely/never eat between meals	71 (15.7%)	60 (15.3%)	28 (12.2%)	66 (17.7%)	37 (15.2%)	68 (16.3%)	63 (14.7%)	131 (15.5%)
Other responses	381 (84.3%)	333 (84.7%)	201 (87.8%)	306 (82.3%)	207 (84.8%)	349 (83.7%)	365 (85.3%)	714 (84.5%)
*X*^*2*^*; p value*	*0.031; 0.86*		*3.32; 0.19*			*0.41; 0.52*		
Frequency of teeth brushing								
> 2ce a day	216 (47.8%)	227 (57.8%)	110 (48.0%)	195 (52.4%)	138 (56.6%)	201 (48.2%)	242 (56.5%)	443 (52.4%)
< 2ce a day	236 (52.2%)	166 (42.2%)	119 (52.0%)	177 (47.6%)	106 (43.4%)	216 (51.8%)	186 (43.5%)	402 (47.6%)
*X*^*2*^*; p value*	*8.38; 0.004*		*3.44; 0.18*			*5.89; 0.02*		
Frequency of teeth flossing								
> 2ce a day	92 (20.4%)	96 (24.4%)	49 (21.4%)	78 (21.0%)	61 (25.0%)	68 (16.3%)	120 (28.0%)	188 (22.2%)
< 2ce a day	360 (79.7%)	297 (75.6%)	180 (78.6%)	294 (79.0%)	183 (75.0%)	349 (83.7%)	308 (72.0%)	657 (77.8%)
*X*^*2*^*; p value*	*2.02; 0.16*		*1.52;0.47*			*16.80; < 0.001*		
Use of fluoride containing toothpaste								
Always	246 (62.2%)	246 (62.6%)	129 (56.3%)	236 (63.4%)	162 (66.4%)	233 (55.9%)	294 (68.7)	527 (62.4%)
Others	171 (37.8%)	147 (37.4%)	100 (43.7%)	136 (36.6%)	82 (33.6%)	184 (44.1%)	134 (31.3)	318 (37.6%)
*X*^*2*^*; p value*	*0.02; 0.90*		*5.42; 0.07*			*14.78; < 0.001*		
Frequency of dental check-ups								
6–12 monthly	123 (27.2%)	144 (36.6%)	87 (38.0%)	114 (30.7%)	66 (27.1%)	84 (20.1)	183 (42.8%)	267 (31.6%)
Others	329 (72.8%)	249 (63.4%)	142 (62.0%)	258 (69.4%)	178 (73.0%)	333 (79.9)	245 (57.2%)	578 (68.4%)
*X*^*2*^*; p value*	*8.65; 0.003*		*6.82; 0.03*			*49.97; < 0.001*		

There were sex, age and state differences in the responses on frequency of dental check-ups. There also were State differences in the responses on frequency of tooth brushing, teeth flossing, and use of fluoride-containing toothpaste, as illustrated in Table 1. Significantly more females than males (*p* = 0.003), more 10–14-year-olds than 15–24-year-olds (*p* = 0.03), and more respondents from Lagos than Osun State (*p* < 0.001) indicated that dental check-up visits should be every 6–12 months. Also, significantly more respondents from Lagos than Osun State knew of the need to brush the teeth twice a day or more (*p* = 0.02), floss daily (*p* < 0.001), and regularly use fluoride-containing toothpaste for tooth brushing (*p* < 0.001).

### Oral hygiene practice

Seventy-five (8.88%) respondents had good oral hygiene practices. The majority of the respondents cleaned their teeth less often than twice a day (93.6%), did not use dental floss (95.6%), consumed sugar between meals (85.0%), and did not have an annual dental check-up (94.0%). Table 2 lists the proportion of respondents for each item of oral hygiene practice.

**Table 2:** Oral hygiene practices of SIYP in Lagos and Osun State, Nigeria (*N* = 845)

Variables	Sex		Age			Residence		Total *N* = 845
	Male (*n* = 452)	Female (*n* = 393)	10–14 years (*n* = 229)	15–19 years (*n* = 372	20–24 years (*n* = 244)	Lagos (*n* = 428)	Osun (*n* = 417)	
Sugar intake in-between meals								
Rarely/never eat between meals	68 (15.0%)	63 (16.0%)	26 (11.4%)	67 (18.0%)	38 (15.6%)	70 (16.4%)	61 (14.6%)	131 (15.5%)
Other responses	384 (85.0%)	330 (84.0%)	203 (88.7%)	305 (82.0%)	206 (84.4%)	358 (83.6%)	356 (85.4%)	714 (84.5%)
*X*^*2*^*; p value*	*0.16; 0.69*		*3.32; 0.19*			*0.48;0.49*		
Frequency of teeth brushing								
> 2ce a day	29 (6.4%)	31 (7.9%)	21 (9.2%)	20 (5.4%)	19 (7.8%)	49 (11.5%)	11 (2.6%)	60 (7.1%)
< 2ce a day	423 (93.6%)	362 (92.1%)	208 (90.8%)	352 (94.6%)	225 (92.2%)	379 (88.6%)	406 (97.4%)	785 (92.9%)
*X*^*2*^*; p value*	*0.69; 0.41*		*3.44; 0.18*			*24.86; < 0.001*		
Frequency of teeth flossing								
> 2ce a day	20 (4.4%)	15 (3.8%)	8 (3.5%)	15 (4.0%)	12 (4.9%)	22 (5.1%)	13 (3.1%)	35 (4.1%)
< 2ce a day	432 (95.6%)	378 (96.2%)	221 (96.5%)	357 (96.0%)	232 (95.1%)	406 (94.9%)	404 (96.9)	810 (95.9%)
*X*^*2*^*; p value*	*0.20; 0.66*		*0.62; 0.73*			*2.17; 0.14*		
Use of fluoride containing toothpaste								
Always	134 (29.6%)	108 (27.5%)	104 (45.4%)	95 (25.5%)	43 (17.6%)	175 (40.9%)	67 (16.1%)	242 (28.6%)
Others	318 (70.4%)	285 (72.5%)	125 (54.6%)	277 (74.5%)	201 (82.4%)	253 (59.1%)	350 (83.9%)	603 (71.4%)
*X*^*2*^*; p value*	*0.48; 0.49*		*47.77; < 0.001*			*63.67; < 0.001*		
Frequency of dental check-up								
6–12 monthly	27 (5.9%)	36 (9.2%)	24 (10.5%)	23 (6.2%)	16 (6.6%)	44 (10.3%)	19 (4.6%)	63 (7.5%)
Others	425 (94.0%)	357 (90.8%)	205 (89.5%)	349 (93.8%)	228 (93.4%)	384 (89.7%)	398 (95.4%)	782 (92.5%)
*X*^*2*^*; p value*	*3.09; 0.08*		*4.20; 0.12*			*10.03; < 0.002*		

There were residential-location differences in the responses on frequency of tooth brushing, use of fluoride-containing toothpaste, and frequency of dental check-ups. There also were age differences in the responses on use of fluoride-containing toothpaste, as illustrated in Table 3. Significantly more respondents in Lagos than in Osun State gave correct responses on frequency of tooth brushing (*p* < 0.001), use of fluoride-containing toothpaste (*p* < 0.001), and frequency of dental check-ups (*p* = 0.002). Also, more 10–14-year-olds than those 15–24-years-old gave appropriate responses to the use of fluoride-containing toothpaste (*p* < 0.001).

**Table 3:** Factors associated with oral hygiene practices among SIYP in Osun and Lagos States, Nigeria (*N* = 845)

Variables	Good oral hygiene practices (*n* = 75)	Poor oral hygiene practices (*n* = 770)	X^2^	*p* value	Total (*N* = 845)
Sex					
Male	36 (8.0%)	416 (92.0%)	1.00	0.32	452 (53.5%)
Female	39 (9.9%)	354 (90.1%)			393 (46.5%)
Age					
10–14 years	27 (11.8%)	202 (88.2%)			229 (27.1%)
15–19 years	31 (8.3%)	341 (91.7%)	3.64	0.16	372 (44.0%)
20–24 years	17 (7.0%)	227 (93.0%)			244 (28.9%)
Residence					
Osun	19 (4.6%)	398 (95.4%)	19.0	< 0.001	417 (49.3%)
Lagos	56 (13.1%)	372 (86.9%)			428 (50.7%)
Ever attended school					
No	8 (12.3%)	57 (87.7%)	1.03	0.31	65 (7.7%)
Yes	67 (8.6%)	713 (91.4%)			780 (92.3%)
Marital status					
Single	69 (9.0%)	702 (91.1%)	0.06	0.81	771 (91.2%)
Married	6 (8.1%)	68 (91.9%)			74 (8.8%)
Oral hygiene knowledge					
Poor	22 (3.8%)	559 (96.2%)	59.55	< 0.001	581 (68.8%)
Good	53 (20.1%)	211 (79.9%)			264 (31.2%)
Water collection and storage					
Poor	36 (6.7%)	501 (93.3%)	8.59	< 0.003	537 (63.6%)
Good	39 (12.7%)	269 (87.3%)			308 (36.4%)
Handwashing					
Poor	54 (8.5%)	581 (91.5%)	0.44	0.51	635 (75.1%)
Good	21 (10.0%)	189 (90.0%)			210 (24.9%)
Sanitation					
Poor	47 (8.2%)	530 (92.0%)	1.20	0.27	577 (65.1%)
Good	28 (10.5%)	240 (89.5%)			268 (34.9%)
Waste disposal					
Poor	41 (8.8%)	424 (91.2%)	0.04	0.94	465 (55.0%)
Good	34 (9.0%)	346 (91.0%)			380 (45.0%)

*estimates derived from Fisher’s exact test

Table 3 lists the three factors significantly associated with oral hygiene practices. More respondents in Lagos than in Osun State (13.1% vs 4.6%; *p* < 0.001); more respondents with good than poor oral hygiene knowledge (9.6% vs 0.0%; *p* < 0.01); and more respondents with good than poor water collection and storage (12.4% vs 4.7%; *p* < 0.001) had good oral hygiene practice.

### Association between oral hygiene practice and oral hygiene knowledge

Table 4 shows the associations between oral hygiene practice and oral hygiene knowledge. There was a significant association between knowledge and the practices of frequency of tooth cleaning, use of fluoride-containing toothpaste, use of dental floss, consumption of sugar between meals, and frequency of dental check-ups (*p* < 0.001 respectively). There were also significant associations between the knowledge on the frequency of tooth brushing and the consumption of sugar between meals (*p* = 0.01), use of fluoridated toothpaste (*p* = 0.004), and frequency of dental check-ups (*p* = 0.001). The knowledge on frequency of dental check-ups was significantly associated with appropriate practices on the consumption of sugar between meals (*p* < 0.001) and use of fluoridated toothpaste (*p* < 0.001).

**Table 4:** Association between oral hygiene knowledge and oral hygiene practices of SIYP in Lagos and Osun State, Nigeria (*N* = 845)

		Oral Hygiene Practices							
Oral Hygiene Knowledge	Variables	Sugar intake in-between meals		Frequency of teeth cleaning		Use of fluoride containing toothpaste		Frequency of check-ups	
		Rarely or never eat between meals	Other responses	> 2ce a day	<2ce a day	Always	Others	Every 6–12 months	Others
	Sugar intake in-between meals								
	Rarely or never eat between meals	109 (83.2%)	22 (16.8%)	7 (5.3%)	124 (94.7%)	36 (27.5%)	95 (72.5%)	7 (5.3%)	124 (94.7%)
	Other responses	22 (3.1%)	692 (96.9%)	53 (7.4%)	661 (92.6%)	206 (28.9%)	508 (71.1%)	56 (7.8%)	658 (92.2%)
	*X*^*2*^*; p value*	*542.49; < 0.001*		*0.73; 0.39*		*0.11; 0.08*		*1.00; 0.32*	
	Frequency of teeth cleaning								
	> 2ce a day	83 (18.7%)	360 (81.3%)	50 (11.3%)	393 (88.7%)	146 (33.0%)	297 (67.0%)	46 (10.4%)	397 (89.6%)
	<2ce a day	48 (11.9%)	354 (88.1%)	10 (2.5%)	392 (97.5%)	96 (23.9%)	306 (76.1%)	17 (4.2%)	385 (95.8%)
	*X*^*2*^*; p value*	*7.43; 0.01*		*24.74; < 0.001*		*8.50; 0.004*		*11.57; 0.001*	
	Frequency of use of fluoride containing toothpaste								
	Always	84 (15.9%)	443 (84.1%)	42 (7.9%)	485 (92.0%)	231 (43.8%)	296 (56.2%)	45 (8.5%)	482 (91.5%)
	Others	47 (14.8%)	271 (85.2%)	18 (5.7%)	300 (94.3%)	11 (3.5%)	307 (96.5%)	18 (5.7%)	300 (94.3%)
	*X*^*2*^*; p value*	*0.20; 0.65*		*1.60; 0.21*		*158.18; < 0.001*		*2.38; 0.12*	
	Frequency of dental check-ups								
	Every 6–12 months	67 (25.1%)	200 (74.9%)	23 (8.6%)	244 (91.4%)	97 (36.3%)	170 (63.7%)	60 (22.5%)	207 (77.5%)
	Others	64 (11.1%)	514 (88.9%)	37 (6.4%)	541 (93.6%)	145 (25.1%)	433 (74.9%)	3 (0.5%)	575 (99.5%)
	*X*^*2*^*; p value*	*27.41; < 0.001*		*1.36; 0.24*		*11.30; 0.001*		*127.57; < 0.001*	

### Indicators for good oral hygiene practices

Table 5 reports on the outcome of the logistic regression analysis identifying indicators for good oral hygiene practices. Before adjustment of the model, the only indicator for good oral hygiene was good water collection and storage; respondents who had good water collection and storage were more likely to have good oral hygiene practices (OR: 2.01; 95% CI: 1.24–3.24; *P* < 0.01). After adjustment, good oral hygiene knowledge, good water collection and storage, and State of residence were significant indicators of good oral hygiene practices. SIYP who had good oral hygiene knowledge (AOR: 6.16; 95% CI: 3.54–10.72; *P* < 0.001); and good water collection and storage practices (AOR: 1.78; 95% CI: 1.09–2.92; *P* = 0.02) were more likely to have good oral hygiene practices. Also, those residing in Lagos (AOR: 2.38; 95% CI: 1.32–4.31; *P* = 0.004) were more likely to have good oral hygiene than those residing in Osun State.

**Table 5:** Indicators for good oral hygiene among SIYP in Osun and Lagos States, Nigeria (*N* = 845)

Variables	Model 1			Model 2		
	OR	95% Confidence interval	*P*-value	AOR	95% Confidence interval	*P*-value
Water collection and storage						
Poor	1.00	1.00	–	1.00	1.00	–
Good	1.91	1.18–3.10	0.01	1.84	1.09–3.09	0.021
Handwashing						
Poor	1.00	1.00	–	1.00	1.00	–
Good	1.10	0.64–1.90	0.73	0.83	0.45–1.52	0.55
Sanitation						
Poor	1.00	1.00	–	1.00	1.00	–
Good	1.17	0.70–1.96	0.76	1.16	0.67–2.03	0.59
Waste disposal						
Poor	1.00	1.00	–	1.00	1.00	–
Good	1.32	0.74–2.37	0.85	1.17	0.62–2.19	0.63
Oral hygiene knowledge						
Poor				1.00	1.00	–
Good				6.16	3.54–10.72	< 0.001
Sex						
Male				1.00	1.00	–
Female				0.93	0.55–1.58	0.80
Age						
10–14 years				1.00	1.00	–
15–19 years				0.80	0.44–1.45	0.47
20–24 years				0.61	0.30–1.27	0.19
Residence						
Osun				1.00	1.00	
Lagos				2.38	1.32–4.31	0.004
Ever attended school						
No				1.00	1.00	–
Yes				0.58	0.23–1.48	0.26
Marital status						
Single				1.00	1.00	–
Married				1.01	0.35–2.95	0.98

## Discussion

To the best of our knowledge, this is the first study to highlight the association between WASH variables and oral hygiene; and to describe the oral hygiene knowledge and practices of SIYP in Nigeria. Good water collection and storage was associated with good oral hygiene practices. We identified that although the oral hygiene knowledge of SIYP was better than their oral hygiene practice, and that most respondents had poor oral hygiene practices, oral hygiene knowledge significantly correlated with oral hygiene practices and was an indicator of good oral hygiene practices (no SIYP with poor oral hygiene knowledge had good oral hygiene). In addition, good oral hygiene knowledge and good water collection and storage were significantly associated with good oral hygiene practices; and the oral hygiene practices of SIYP residing in cosmopolitan Lagos State were better than that of respondents residing in the less urbanized Osun State.

One strength of this study is the large sample size that made it possible to conduct subgroup analysis. We avoided the use of a summative index to describe oral health practice and WASH because the index may have obscured item-level distinctions and decreased the predictive value of the construct [32]. This decision enabled us to identify how specific WASH items were associated with specific oral hygiene practices. Our study on the association between WASH and oral health is important also because it can help programmers identify ways to leverage the successful and resourced WASH program to improve oral hygiene in developing countries where oral health epidemics are growing [33]. We also add to the growing body of knowledge on WASH [34].

Good oral hygiene practices, such as tooth brushing and the use of toothpaste, requires access to water. When access to water is poor, adolescents may forgo tooth brushing because it presents no immediate risks for their aesthetics. Adolescents prioritize good body image [35], so they may use collected and stored water for other hygiene purposes other than oral health. Water access is a major problem in Nigeria, with only 26.5% of the population having access to improved drinking water sources [33], a situation that is not improving [36]. Investments in improving water supply and storage may result in improved oral hygiene for SIYP, with the potential of also improving the health and wellbeing of SIYP. Until now, there has been little or no discussions on the impact of WASH on oral health. This study reveals the potential value of WASH programs for oral health and for adolescents and young persons. It also highlights the feasibility of the inclusion of oral health outcomes as an indicator of the success of the programs.

Some WASH indicators have been associated with oral hygiene practices. For example, in the Philippines, handwashing campaigns were run in conjunction with tooth brushing and deworming campaigns for children [37]. We found no significant association between handwashing and oral hygiene practices for SIYP in Nigeria. This may suggest that collaborative WASH and oral health campaigns should focus on different WASH and oral hygiene themes for different populations according to age and culture/norms.

This study also highlights the need to improve the oral hygiene practice of SIYP. Good oral hygiene knowledge, a predictor of good oral hygiene practices in this study, may be a useful strategy. The need to improve oral hygiene knowledge may not be peculiar to SIYP, as other studies conducted with adolescents in Nigeria show they have comparable oral hygiene practices [38]; dental service utilization is poor [48]; a high proportion of the youths consume refined carbohydrate between meals; and a high proportion brush less than twice a day [36, 39]. Associations between oral hygiene knowledge and practices have been reported in other populations [40], and, as in this study, practice is always poorer than knowledge. Better access of Nigerian SIYP to oral hygiene education may significantly improve the proportion of this population that practices good oral hygiene, which may be a simple route to improved quality of life, wellbeing and life expectancy.

Finally, residential location is significantly associated with oral hygiene practices of SIYP. Lagos, in Lagos State, where the study participants were recruited, is a cosmopolitan city. Access to information, oral health care services and improved water collection and storage that affects oral hygiene practices, are more likely accessible in cosmopolitan Lagos State than in hinterland Osun State: in 2015, 82% of urban dwellers and 54% of rural dwellers had access to basic water in Nigeria [41]. The difference in the oral hygiene of SIYP in Lagos and Osun State, and the national profile on water access may be proof of the increased risk of SIYP residing in hinterlands in Nigeria, to poorer oral health than their peers residing in cosmopolitan areas. This hypothesis, however, is unproven and needs investigation.

This study has some limitations. First, is the inherent limitation of the cross-sectional design of the study. We were unable to establish a cause-effect relationship between WASH and poor oral hygiene practices. Second, there may be multiple confounders that were not identified and controlled for; our ability to identify these confounders is limited since almost no studies have been conducted on the subject matter. We, however, controlled for some possible confounders, using logistic regression. Third, the study may suffer from social desirability response bias when questioned on oral health practices [42]. However, we felt this impact was minimal as there was a large difference in the responses to oral hygiene knowledge and oral hygiene practices. Fourth, we did not distinguish between participants who make a living on the street but live in a home and have family contact, from those whose permanent place of residence is the street and have no other contacts or are completely abandoned [43]. This information was important as this may be a confounder for hygiene practices. The study data was collected from cities in Southwest Nigeria limiting its generalizability to Nigeria in view of the differences in the social contexts of the lives of people in northern and eastern Nigeria, which may impact the variables being studied. Most of the study participants were recruited through direct recruitment with implications for the sample representativeness also. Despite these limitations, the study provided useful new information and helped generate new study hypotheses that can be pursued through primary data collection.

## Conclusion

For SIYP in Nigeria, the residential location further increases the vulnerability to poor oral hygiene practices. The association between access to good water collection and storage and good oral hygiene practices highlights the possible indirect impact the WASH program can have on improving health through improving oral hygiene practices. Although many WASH programs have focused on improving the health of children, this study indicates that a WASH indicator is associated with oral health practices in adolescents; extending WASH programs to include adolescents in Nigeria should be considered. The study findings also suggest the need to include a monitoring indicator of oral hygiene practice in the WASH monitoring and evaluation framework.

## Data Availability

The datasets used and analysed during the current study are available from the corresponding author on reasonable request.

## Abbreviations

AOR: Adjusted odds ratio
CEP: Community engagement program
CI: Confidence interval
OR: Odds ratio
OVC: Orphaned and vulnerable children
SIYP: Street-involved young people
UNHCR: United Nations high commission for refugees
WASH: Water, sanitation and hygiene
